# Examining Social Touch in Early-Life Stress

**DOI:** 10.1192/bjo.2024.195

**Published:** 2024-08-01

**Authors:** Lena Lim

**Affiliations:** ASTAR, Singapore, Singapore

## Abstract

**Aims:**

Social contact is crucial for both immediate and later development of adaptive social and emotional behaviour. Tactile experiences during childhood influence the development of the social brain and frequent affectionate touch is associated with secure attachment style. Social touch is an important form of social interaction and plays a significant role in the formation and maintenance of relationships in humans across development, where the hedonic properties of touch are involved in improving the quality of life. However, relatively less research attention has focused on social touch experiences in individuals with a history of early-life interpersonal stress, particularly childhood maltreatment.

**Methods:**

Social touch pleasantness ratings using a newly developed Social Touch task and attitudes about a variety of social touch behaviours using the Social Touch Questionnaire (STQ) were examined in 40 age- and gender-matched young adults (23 childhood maltreatment, 17 controls).

**Results:**

The childhood maltreatment group had significantly lower STQ score than the control group, where lower STQ score was furthermore correlated with higher severity of maltreatment, particularly physical neglect. For the social touch task, females who experienced childhood maltreatment had significantly lower mean pleasantness ratings for positive social touch than their male counterparts, and these differences were mainly in response to touch given by stranger and friend of opposite gender.

**Conclusion:**

These preliminary results show that early-life interpersonal stress from caregivers may potentially influence touch processing and pleasantness, particularly for females, and there is a need to further explore the effects of different touch giver role (e.g. friend, stranger, partner).

